# Design of phononic crystal using open resonators as harmful gases sensor

**DOI:** 10.1038/s41598-023-36216-y

**Published:** 2023-06-08

**Authors:** Zaky A. Zaky, M. A. Mohaseb, Ahmed S. Hendy, Arafa H. Aly

**Affiliations:** 1 TH-PPM Group, Physics Department, Faculty of Science, Beni-Suef University, Beni Suef, 62521 Egypt; 2 Department of Physics, College of Applied Sciences, Umm Al-Qura University, Mecca, Saudi Arabia; 3 Department of Computational Mathematics and Computer Science, Institute of Natural Sciences and Mathematics, Ural Federal University, 19 Mira St., Yekaterinburg, Russia 620002

**Keywords:** Sensors and biosensors, Acoustics

## Abstract

This paper investigates the ability to use a finite one-dimensional phononic crystal composed of branched open resonators with a horizontal defect to detect the concentration of harmful gases such as CO_2_. This research investigates the impact of periodic open resonators, defect duct at the center of the structure, and geometrical parameters such as cross-sections and length of the primary waveguide and resonators on the model's performance. As far as we know, this research is unique in the sensing field. Furthermore, these simulations show that the investigated finite one-dimensional phononic crystal composed of branched open resonators with a horizontal defect is a promising sensor.

## Introduction

Massively producing pollutants in the air has threatened human health, the environment, and global biological ecosystems in recent years^1,2^. So, detecting harmful gases to human health, such as CO_2_, NO_2_, NH_3_, etc., piqued people's interest in protecting humans and the environment^3–6^. As a result, numerous optical studies have been done on detecting toxic gaseous using two-dimension nanostructured materials, such as porous materials^5,7^ and graphene^8,9^. In addition, fluorescent, chemical, electrochemical, photonic crystal, and mass-sensitive are common gas sensors^10–13^.

Phononic crystals (PnCs) are periodic artificial materials^14–16^. PnCs have sparked considerable interest in various biosensing and chemical applications. PnCs can confine acoustic or elastic waves by creating stop frequency bands or phononic bandgaps (PnBGs) to propagate through them^17,18^. Acoustic properties of materials, such as viscosity, density, speed of sound, elastic moduli, etc., can be probed by propagating the acoustic wave inside^19^. One-dimensional PnC (1D-PnC) sensors are resonant detectors. The main operating concept of 1D-PnC sensors is the multiple Bragg scattering of acoustic waves at each interface between two mediums with different acoustic impedance to produce a standing wave. The frequency of the PnBG depends on the traveling wave's acoustic speed and the structure's geometrical dimensions. Most 1D-PnC sensors are based on breaking the periodicity at the center of the structure, resulting in a resonant peak inside the PnBG. Adding this defect at the center of the structure confines a specific frequency called resonant frequency.

In traditional PnCs, continuity of flux and pressure are considered along the main direction of propagation. Recently, locally resonant elements have attracted attention in the field of periodic structures. However, lateral elements or resonators that depend on the change of pressure or flux stability in other paths can be added. These lateral elements can be closed or open ducts. In 2008, El Boudouti et al.^20^ proposed a structure of a slender tube with lateral ducts. The presence of lateral tubes causes the formation of stop bands in the transmittance spectrum. In 2020, Antraoui et al. designed a periodic structure composed of a main duct with open resonators. But utilizing these structures with lateral resonators in gas sensing applications is still lacking.

Recently, gas sensors using PnCs attracted attention due to their advantages. For example, gas sensors using PnCs do not require a recovery time. Besides, as PnC doesn’t contain any electronic component, gas sensors using PnCs can give good measurements in complex environments such as in an explosive environment^21^. Furthermore, the low cost and ease of fabrication of PnC sensors are good advantages^22^.

As far as we know, this research is unique in the gas sensing field. Using branched open resonators enhanced the sensor's performance. Furthermore, these simulations show that the investigated finite one-dimensional phononic crystal composed of branched open resonators with a horizontal defect is a promising sensor. Furthermore, the proposed PnC sensor with branched open resonators can be easily fabricated using low-cost conventional materials.

## Sensor configuration and equations

**Figure 1:**
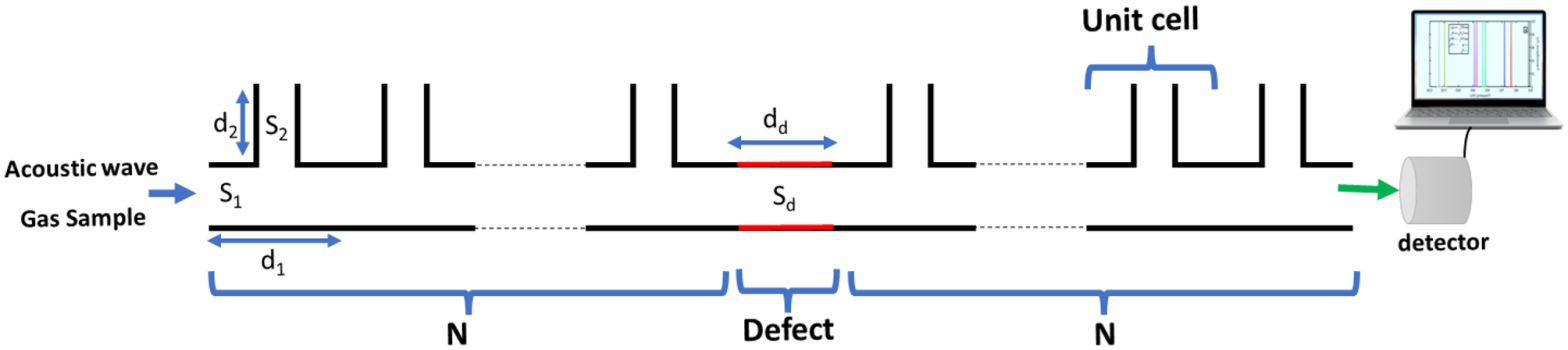
Schematic of the 1D-PnC composed of branched open resonators.

In Fig. 1, a schematic of the 1D-PnC composed of branched open resonators is proposed. The main guide has a cross-section *S*_*1*_ and a thickness *d*_*1*_. The branched open resonators have cross-section *S*_*2*_ and height *d*_*2*_. The proposed 1D-PnC comprises branched-open resonators sensor, and a defect guide sandwiched between two PnCs. The structure will be filled with gas samples containing different concentrations of CO_2_. The plane wave theory can be used for stationary samples inside the sensor, and the effects of temperature gradients, higher-order modes, and viscosity effects are neglected^23^.

The theoretical method used to study the response of the proposed periodic branched open resonators to the incident acoustic waves is called the transfer matrix method (TMM) as the following^23–30^:

1 $$M_{i} = \left[ {\begin{array}{*{20}c} {A_{i} } & {B_{i} } \\ {C_{i} } & {D_{i} } \\ \end{array} } \right]\left[ {\begin{array}{*{20}c} 1 & 0 \\ {y_{R} } & 1 \\ \end{array} } \right]\left[ {\begin{array}{*{20}c} {A_{i} } & {B_{i} } \\ {C_{i} } & {D_{i} } \\ \end{array} } \right],$$

where $$A_{i} = \cos \left( {k\frac{{d_{i} }}{2}} \right), B_{i} = j Z_{i} sin\left( {k\frac{{d_{i} }}{2}} \right), C_{i} = \frac{j}{{Z_{i} }}sin\left( {k\frac{{d_{i} }}{2}} \right), D_{i}$$ = $$A_{i}$$*,* $$k = {\raise0.7ex\hbox{$\omega $} \!\mathord{\left/ {\vphantom {\omega c}}\right.\kern-0pt} \!\lower0.7ex\hbox{$c$}}$$ is the wave number, $$\rho$$ is the density, $$Z_{i} = {\raise0.7ex\hbox{${\rho c}$} \!\mathord{\left/ {\vphantom {{\rho c} {{\text{S}}_{{\text{i}}} }}}\right.\kern-0pt} \!\lower0.7ex\hbox{${{\text{S}}_{{\text{i}}} }$}}$$ is the impedance of each period of the proposed branched open resonators, and $$c$$ is the acoustic speed. The acoustic pressure at the end of the opened lateral chimney is approximately zero, and the acoustic admittance of the acoustic wave ($$y_{R} )$$ is calculated as:

2 $$y_{R} = - j \frac{1}{{Z_{2} }} cot\left( {kd_{2} } \right).$$

For the defect cell:

3 $$M_{i} = \left[ {\begin{array}{*{20}c} {A_{d} } & {B_{d} } \\ {C_{d} } & {D_{d} } \\ \end{array} } \right],$$

where $$A_{d} = \cos \left( {k\frac{{d_{d} }}{2}} \right), B_{d} = j Z_{d} sin\left( {k\frac{{d_{d} }}{2}} \right), C_{d} = \frac{j}{{Z_{d} }}sin\left( {k\frac{{d_{d} }}{2}} \right), D_{d}$$ = $$A_{d}$$*,* and $$Z_{d} = {\raise0.7ex\hbox{${\rho c}$} \!\mathord{\left/ {\vphantom {{\rho c} {{\text{S}}_{{\text{d}}} }}}\right.\kern-0pt} \!\lower0.7ex\hbox{${{\text{S}}_{{\text{d}}} }$}}$$.

Bloch’s theorem is used to plot the dispersion relation of the elementary cell of the 1D-PnC composed of branched open resonators^23^:

4 $${\text{cos}}\left( {Kd} \right) = \cos \left( {kd_{1} } \right) + \frac{M}{2}\sin \left( {kd_{1} } \right)\cot \left( {kd_{2} } \right),$$

where $K$ is the Bloch vector,$d = d_{1} + d_{2}$, $M = \frac{{S_{2} }}{{S_{1} }}$, *k* is the wave vector. The transmission and transmittance of the 1D-PnC composed of branched open resonators are calculated as the following:

5 $$t = \frac{{2\emptyset_{1} }}{{\left( {A_{11} + A_{12} \emptyset_{1} } \right)\emptyset_{1} + \left( {A_{21} + A_{22} \emptyset_{1} } \right)}} ,\;{\text{where}}\;\emptyset_{1} = \frac{1}{z}$$

6 $$T\left( \% \right) = 100*\left| t \right|^{2}$$

## Results and discussions

As an initial condition, the geometrical parameters of the main guide and open resonators of the proposed sensors will be *N* = 10, *d*_*1*_ = 0.6 m, *d*_*2*_ = 0.15 m, *d*_*d*_ = 0.3 m, *S*_*1*_ = 1 m^2^, *S*_*2*_ = 0.75 m^2^, and *S*_*d*_ = *S*_*1*_ m^2^. Table 1 shows the acoustic properties of an air sample at different concentrations of CO_2_. The gradient of the density of the sample from low to high and acoustic speed from high to low with the increase of the CO_2_ concentration ensures that both density and acoustic speed can be considered an indicator of the concentration of CO_2_.

**Table 1:** Acoustic properties of an air sample at different concentrations of CO_2_^31^.

CO_2_ concentration (%)	Density ($\rho$) (Kg/m^3^)	Acoustic speed ($c$) (m/s)
0	1.2047	343
20	1.33162	325.1
40	1.45854	307.6
60	1.58546	290.3
80	1.71238	279.9
100	1.8393	273.4

**Figure 2:**
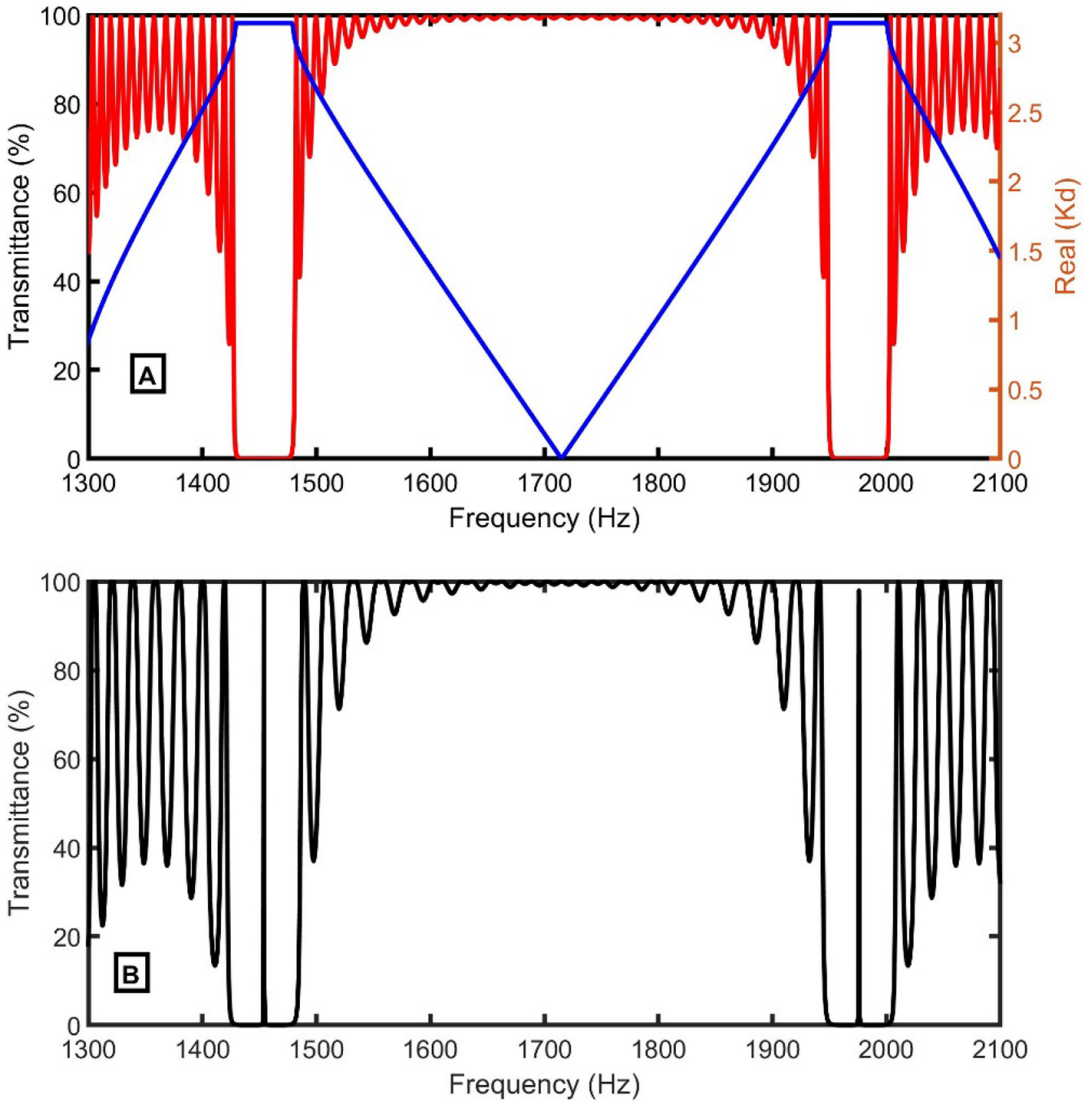
(**A**) The dispersion relation (blue line), the transmittance of the 1D-PnC composed of branched open resonators without defect cell (red line) using air sample (exceed in CO_2_ = 0%), and (**B**) the transmittance with a defect (blue spectrum) using air sample with different CO_2_ concentrations.

The transmittance (red spectra) and dispersion relation (blue spectra) curves versus frequency of the proposed 1D-PnC composed of branched open resonators without defect are plotted and coincided using TMM and Bloch’s theorem in Fig. 2A. In the frequency range of concern, two PhBGs extend from 1429.2 to 1478.1 Hz and from 1950.6 to 2000.6 Hz. The proposed 1D-PnC sensor composed of branched open resonators has the ability to make the PnBG due to the periodic change in the impedance and admittance of propagated acoustic waves inside the structure. By adding a horizontal defect tube sandwiched between two identical 1D-PnCs, a specific frequency of the incident acoustic wave is localized, making a defect peak inside the PnBG. This peak is very sensitive to any change in the mechanical properties of the medium inside the tubes. Considering an additional defect tube with *d*_*d*_ = 0.3 m at the middle of the design and the other geometrical parameters having the same initial values, a resonant peak appears at the center of each PnBG, as clear in Fig. 2B.

**Figure 3:**
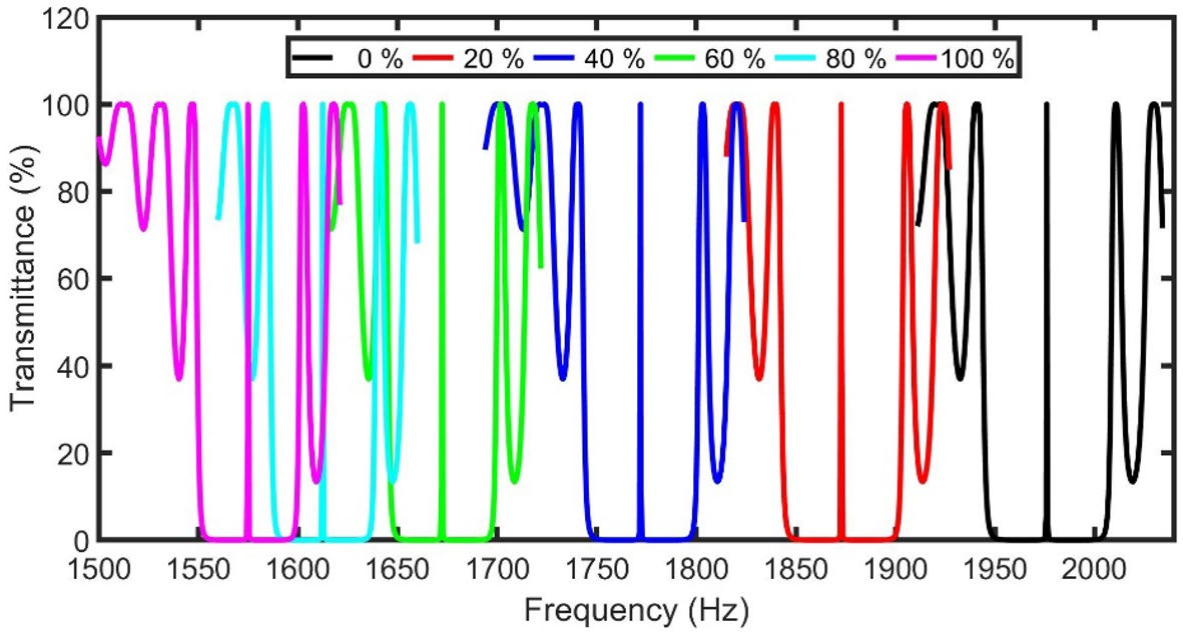
The transmittance of the 1D-PnC sensor composed of branched open resonators with a defect cell using different concentrations of CO_2_.

Any change in the density or acoustic speed of the gas sample due to the change in the CO_2_ concentration will result in a transmittance spectrum and cause a wavelength shift to the resonant peaks and PnBGs, as clear in Fig. 3. The defect peak is redshifted to lower frequencies by increasing the concentration of CO_2_ from 1975.95 Hz (at 0% of CO_2_) to 1872.83 Hz (at 20% of CO_2_), 1772.02 Hz (at 40% of CO_2_), 1672.36 Hz (at 60% of CO_2_), 1612.45 Hz (at 80% of CO_2_), and 1575.00 Hz (at 100% of CO_2_).

The sensitivity, figure of merit (*FoM*), quality factor (*Q*), and detection limit (*LoD*) of the harmful gas’s sensor are used to examine the efficacy of the sensor and can be defined as follows,

7 $$S = \frac{{\Delta f_{R} }}{\Delta c},$$

8 $$FoM = \frac{S}{FWHM},$$

9 $$\begin{array}{*{20}l} {Q = \frac{{f_{R} }}{FWHM}} \hfill \\ \end{array} ,$$

10 $$LoD = \frac{{f_{R} }}{20 S Q},$$

where $\Delta f_{R}$ is the value of the resonant frequency shift with changing the acoustic speed by ($\Delta c$), and *FWHM* is the peak bandwidth. Sensitivity is the change in the position of the defect peak relative to the acoustic speed relative to the pure air sample as a reference. *Q* denotes the resonator's energy loss and is expressed as the ratio of the frequency of the defect peak to the *FWHM*. The sensor's ability to discover the alteration in the resonance frequency is represented by *FoM*^32^. *LoD* denotes the slightest change in the sample that can be detected.

**Figure 4:**
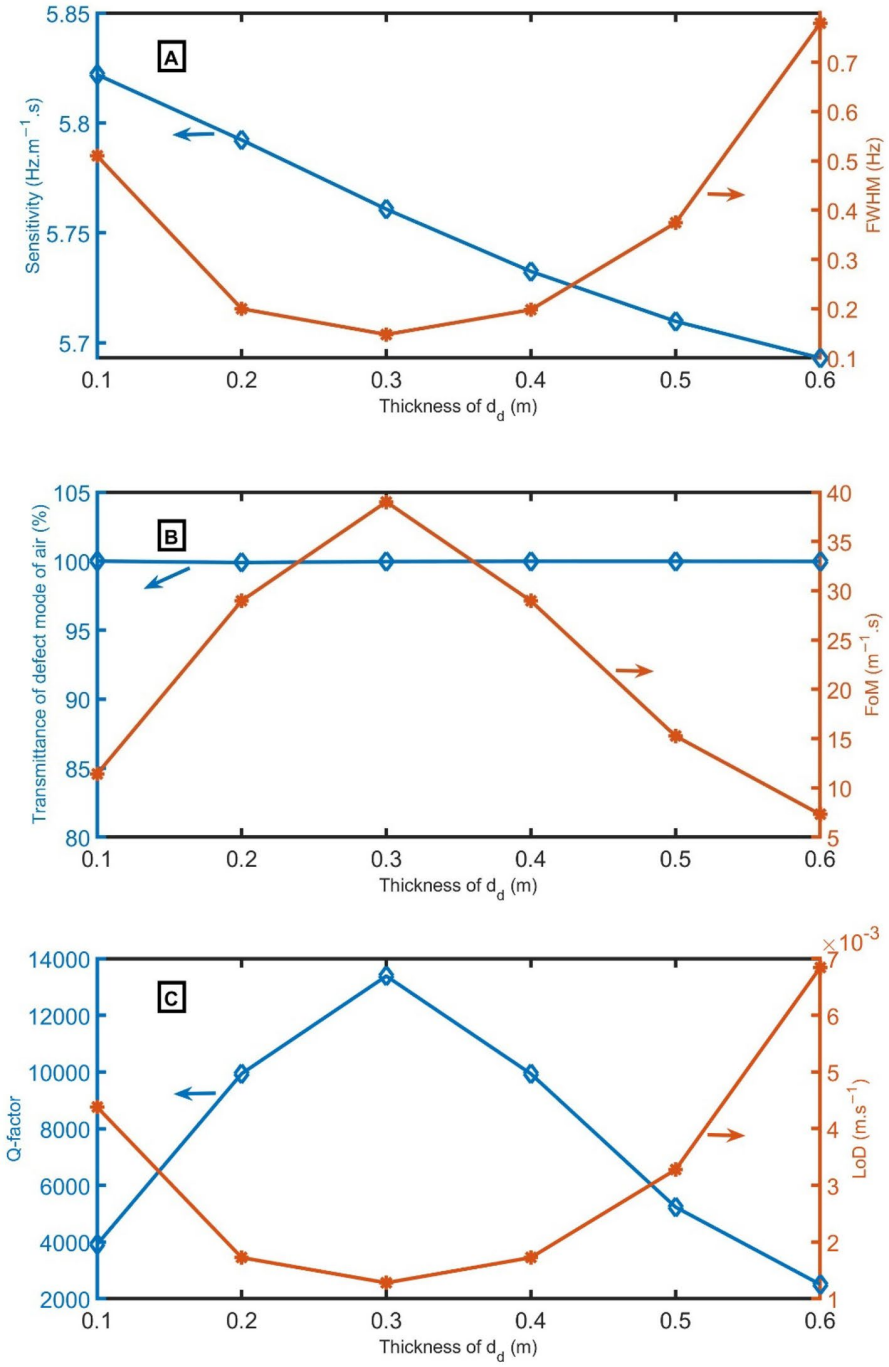
(**A**) *S* and *FWHM*, (**B**) transmittance and *FoM*, and (**C**) *Q* and *LoD* versus the thickness of *d*_*d*_.

Figure 4A–C shows the *S*, *FWHM*, *T, FoM*, *Q,* and *LoD* versus the thickness of *d*_*d*_. Figure 4A clears the sensitivity and *FWHM* versus the incident frequency for the proposed 1D-PnC sensor composed of branched open resonators with a defect cell at different *d*_*d*_ values to select the best value that gives the highest performance. The sensitivity is measured for the proposed sensor at different thicknesses of *d*_*d*_ of 0.1 m, 0.2 m, 0.3 m, 0.4 m, 0.5 m, and 0.6 m. In Fig. 4A, the sensitivity is slightly reduced from 5.82 Hz m^−1^ s to 5.79 Hz m^−1^ s, 5.76 Hz m^−1^ s, 5.73 Hz m^−1^ s, 5.71 Hz m^−1^ s, and 5.69 Hz m^−1^ s with the increase of *d*_*d*_.

Sharp defect peaks with 100% intensity at resonant frequencies of 1996.94 Hz, 1986.81 Hz, 1975.95 Hz, 1966.27 Hz, 1958.48 Hz, and 1952.73 Hz for air sample and frequencies of 1591.73 Hz, 1583.67 Hz, 1575.00 Hz, 1567.29 Hz, 1561.08 Hz, and 1556.49 Hz for CO_2_ sample at thicknesses of 0.1 m, 0.2 m, 0.3 m, 0.4 m, 0.5 m, and 0.6 m, respectively. The right axis of Fig. 4A clears the variations in the *FWHM* of the resonant peak with *d*_*d*_. At *d*_*d*_ = 0.3 m, the *FWHM* has the lowest value of 0.14 Hz. As a result of the behavior of *FWHM*, the *FoM,* and *Q* have the highest values at the same thickness, according to Eqs. (8) and (9). On the other hand, the *LoD* has a minor performance at *d*_*d*_ = 0.3 m.* d*_*d*_ = 0.3 m will be the optimum value. This thickness achieved high performance because the resonant peak is located at the center of the PnBG.

**Figure 5:**
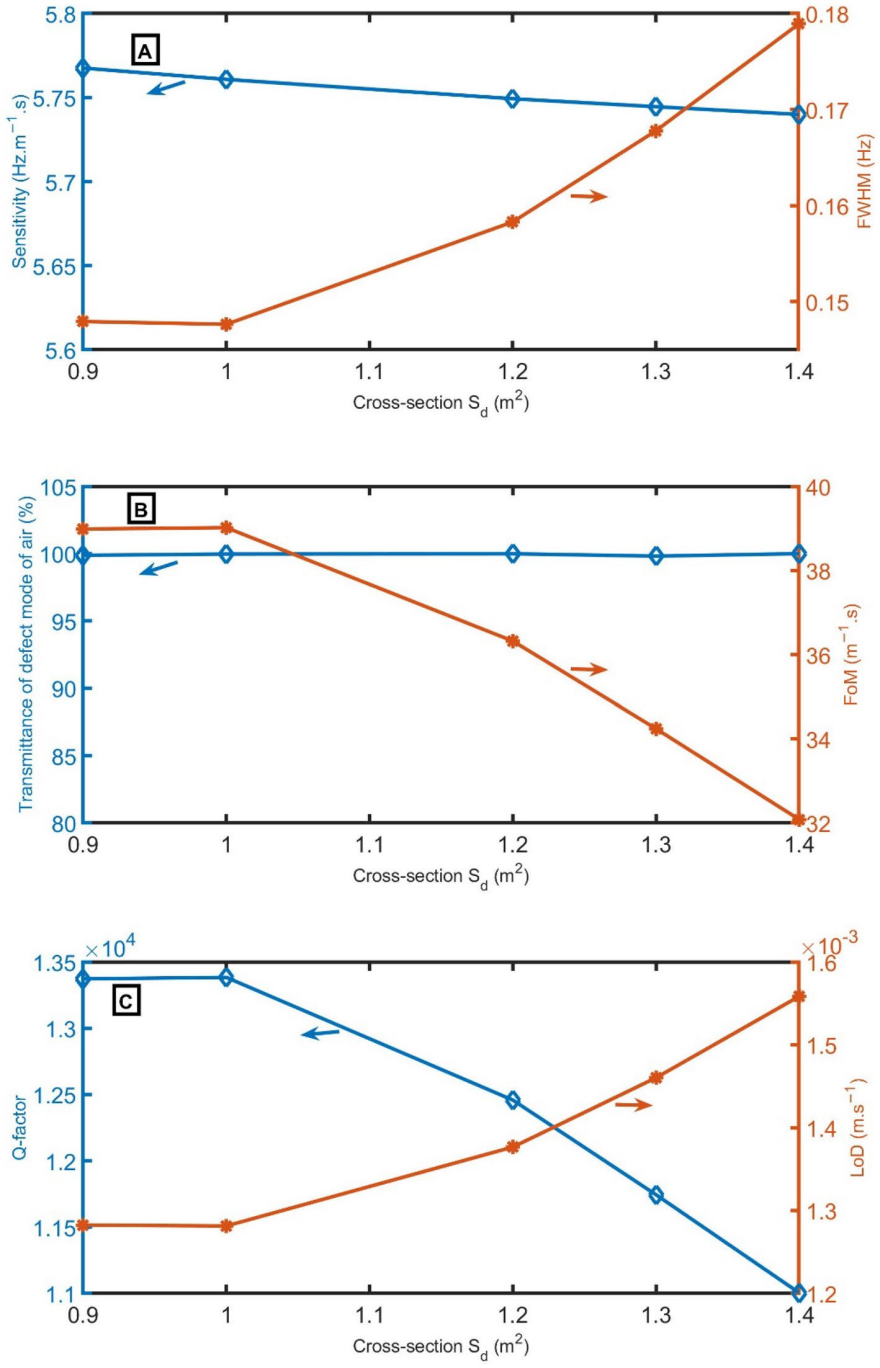
(**A**) *S* and *FWHM*, (**B**) transmittance and *FoM*, and (**C**) *Q* and *LoD* versus the cross-section of *S*_*d*_.

The reliability of the 1D-PnC sensor composed of branched open resonators is investigated by studying the impact of the cross-section of *S*_*d*_ on *S*, *FWHM*, *T, FoM*, *Q,* and *LoD* at different concentrations of CO_2_, as shown in Fig. 5A–C. The defect peak and PnBG exhibit a redshift to lower frequencies as the cross-section of *S*_*d*_ gradually increases. The *S* decreases from 5.77 to 5.74 Hz m^−1^ s as the cross-section of *S*_*d*_ increases from 0.9 to 1.4 m^2^. However, the *FWHM* gradually increases with the cross-section of S_d_. Besides, the *T* of the resonant peak records the highest intensity of (100%). Hence, the *FoM* and *Q* gradually decrease, and *LoD* gradually increases. Depending on the results in Fig. 5A–C, the cross-section of *S*_*d*_ = 1 m^2^ will be used in the following studies.

**Figure 6:**
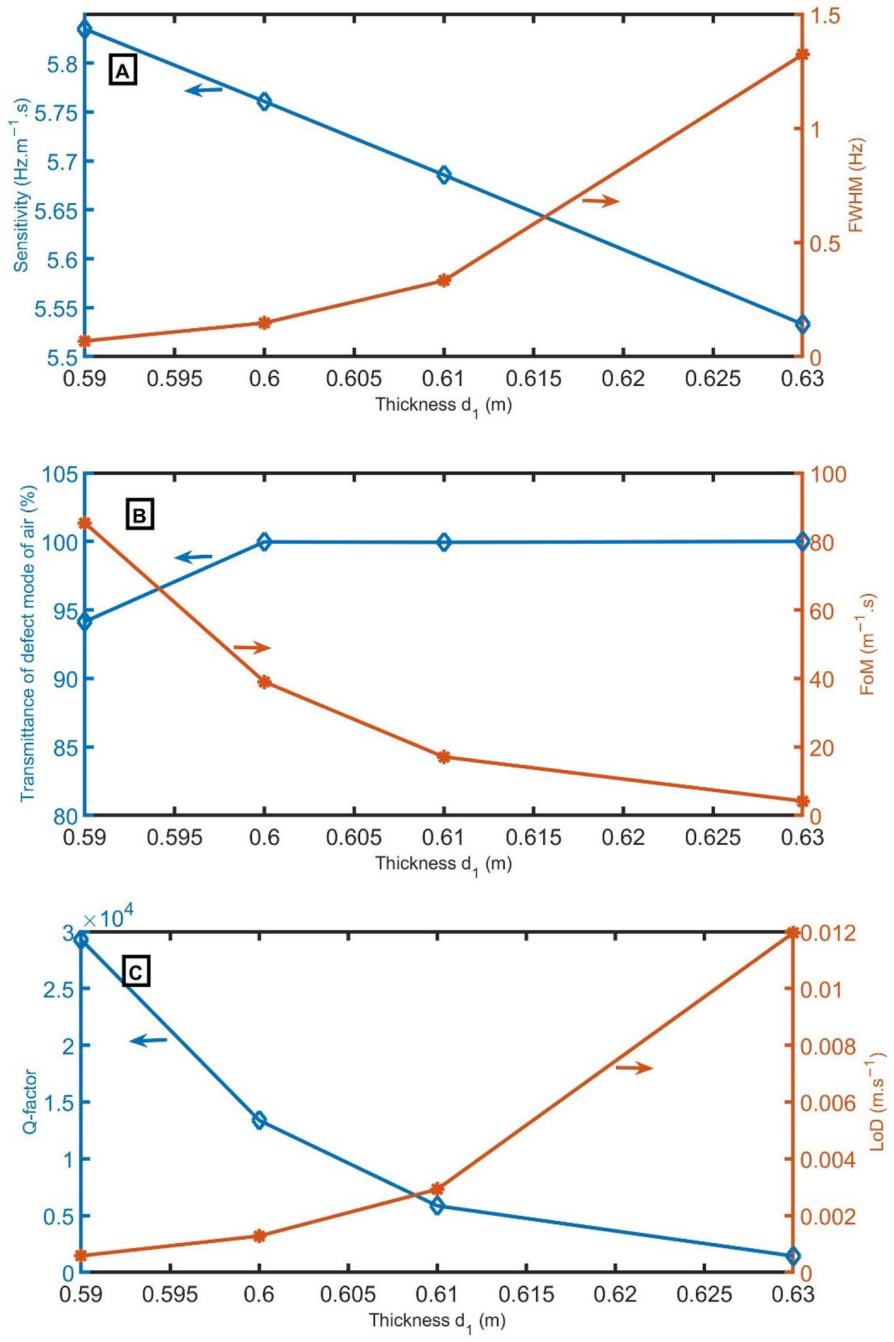
(**A**) *S* and *FWHM*, (**B**) transmittance and *FoM*, and (**C**) *Q* and *LoD* versus the thickness of *d*_*1*_.

As *d*_*1*_ increases from 0.59 m to 0.60 m, 0.61 m, and 0.63 m, the peak of the air sample is redshifted from 2001.36 Hz to 1975.95 Hz, 1950.14 Hz, and 1897.80 Hz, and the peak of the CO_2_ sample is redshifted from 1595.26 Hz to 1575.00 Hz, 1554.42 Hz, and 1512.70 Hz. In Fig. 6A, the sensitivity decreases linearly with increasing *d*_*1*_. On the other hand, *FWHM* gradually increases with increasing *d*_*1*_. The transmittance records intensity above 99.9% for thickness d_1_ higher than 0.59 m, as clear in Fig. 6B,C. Besides, *FoM* and *Q* gradually decrease, and *LoD* gradually increases with increasing *d*_*1*_. Therefore, a thickness of 0.59 m will be optimum.

**Figure 7:**
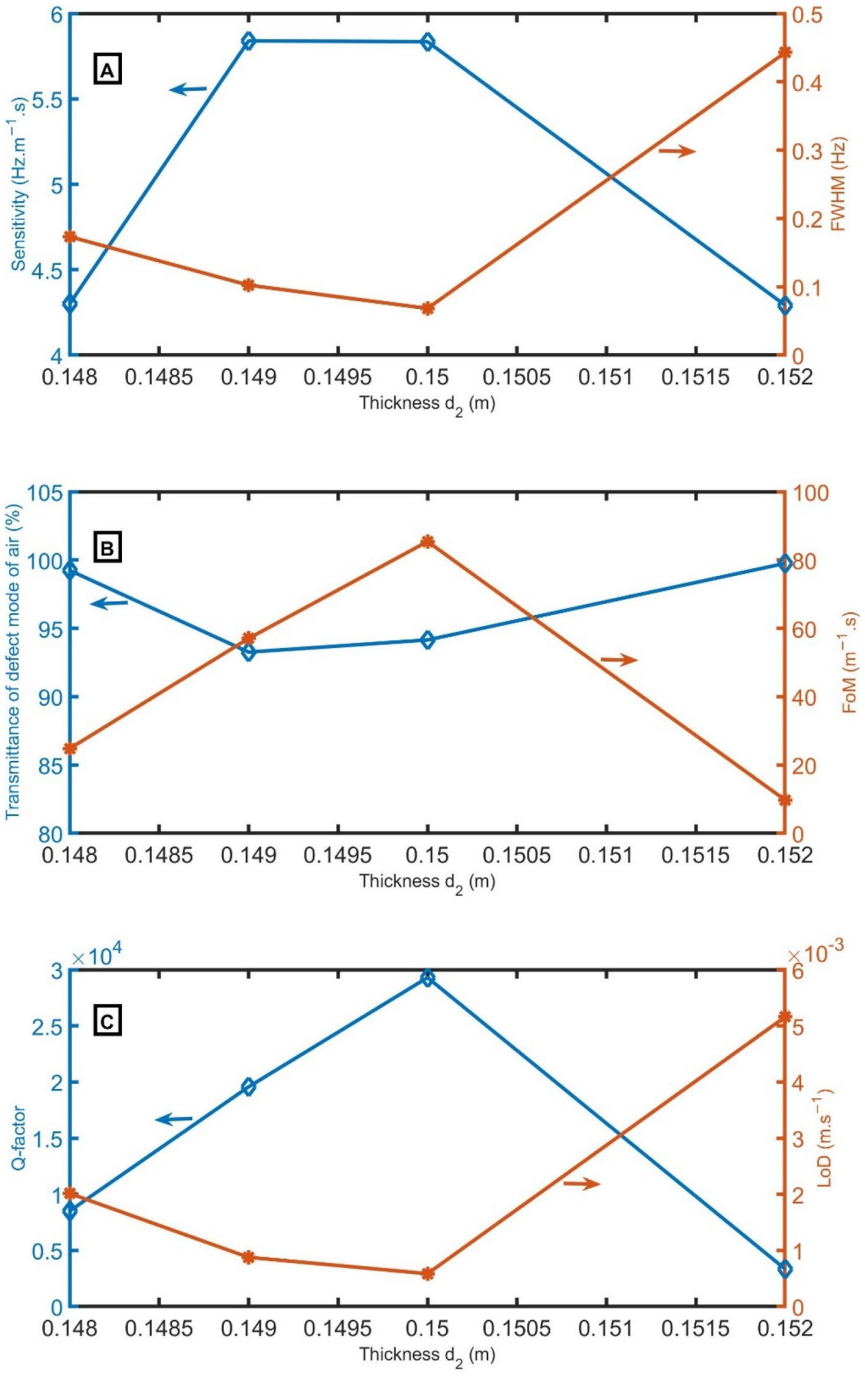
(**A**) *S* and *FWHM*, (**B**) transmittance and *FoM*, and (**C**) *Q* and *LoD* versus the thickness of *d*_*2*_.

Figure 7A clears the sensitivity and *FWHM* versus the incident frequency for the proposed 1D-PnC sensor composed of branched open resonators with a defect cell at different values of *d*_*2*_ to select the best value that gives the highest performance. The sensitivity is measured for the proposed sensor at different thicknesses of *d*_*2*_ of 0.148 m, 0.149 m, 0.15 m, and 0.152 m. In Fig. 7A, the sensitivity is increased from 4.30 Hz m^−1^ s to 5.84 Hz m^−1^ s with the increase of *d*_*2*_ from 0.148 m to 0.149 m. Then, sensitivity slightly decreases to 5.83 Hz m^−1^ s with the increase of *d*_*2*_ to 0.150 m. After that, sensitivity is significantly reduced to 4.29 Hz m^−1^ s with the increase of *d*_*2*_ to 0.152 m. At *d*_*2*_ = 0.150 m, the *FWHM* has the lowest value of 0.068 Hz. The *T* of the resonant peak changes from 99.24% to 93.26%, 94.15%, and 99.76% by changing the thickness of *d*_*2*_ from 0.148 m to 0.149 m, 0.15 m, and 0.152 m. As a result of the behavior of *FWHM* and sensitivity, the *FoM* and *Q* have the highest values at the same thickness, according to Eqs. (8) and (9) and Fig. 7B,C. On the other hand, the *LoD* has the smallest performance at *d*_*2*_ = 0.150 m.* d*_*2*_ = 0.150 m will be the optimum value.

**Figure 8:**
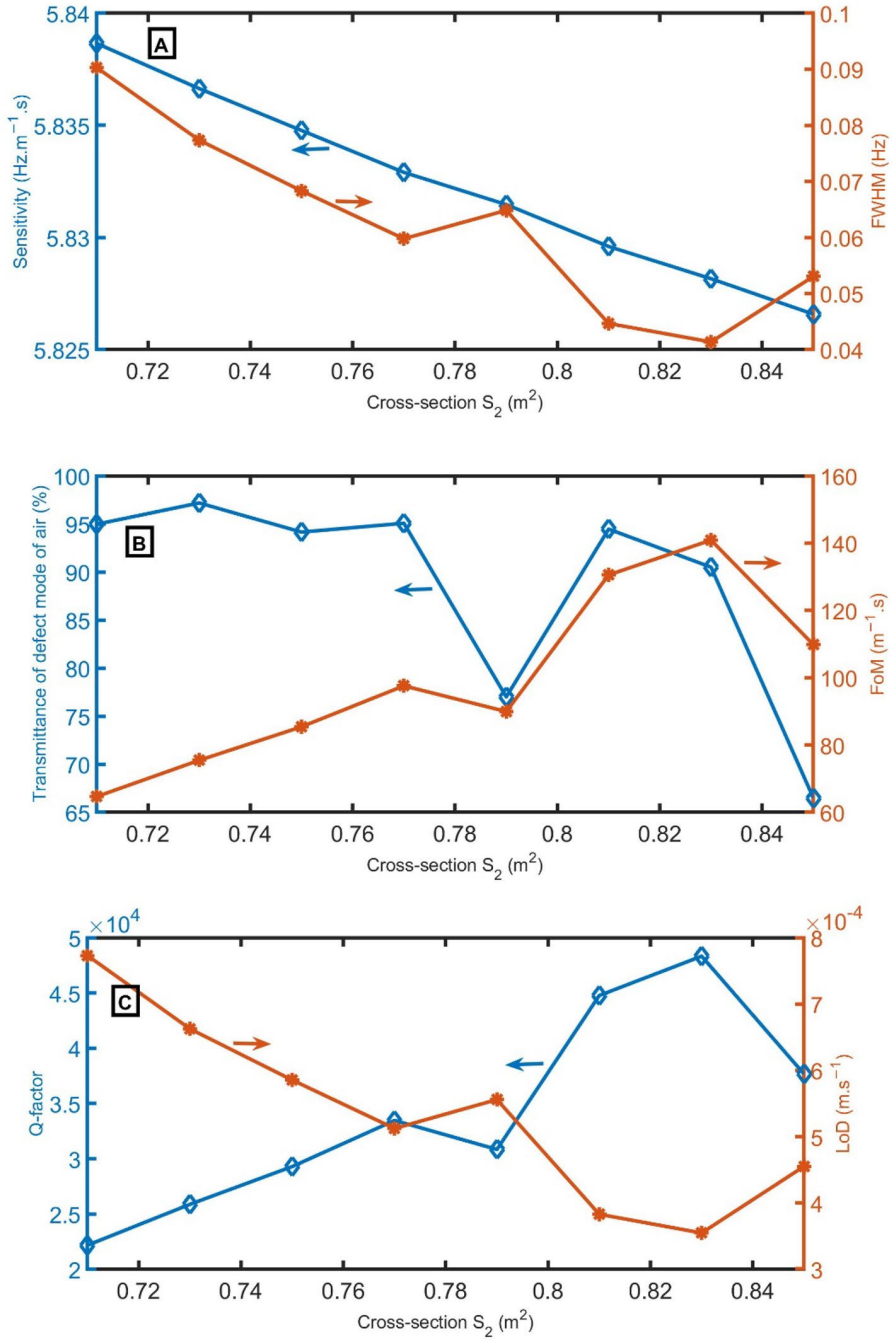
(**A**) *S* and *FWHM*, (**B**) transmittance and *FoM*, and (**C**) *Q* and *LoD* versus the cross-section of *S*_*2*_.

Figure 8A–C shows the variations in *S*, *FWHM*, *T, FoM*, *Q,* and *LoD* with cross-sections S_2_. The defect peak and PnBG exhibit a redshift to lower frequencies as the cross-section of S_2_ gradually increases. The *S* gradually decreases from 5.84 to 5.83 Hz m^−1^ s as the cross-section of *S*_*d*_ increases from 0.71 to 0.85 m^2^. Also, the *FWHM* gradually decreases with the increase of the cross-section of *S*_*2*_ for all selected values of cross-sections except at 0.79 m^2^ and 85 m^2^. At these values (0.79 m^2^ and 85 m^2^), the *FWHM* records a small increase. The *T* of the resonant peak changes from 94.97% to 97.18%, 94.15%, 95.07%, 77.00%, 94.46%, 90.5%, and 66.48% by changing the cross-section of *S*_*2*_ from 0.71 m^2^ to 0.73 m^2^, 0.75 m^2^, 0.77 m^2^, 0.79 m^2^, 0.81 m^2^, 0.83 m^2^, and 0.85 m^2^. *FoM* changes from 64.68 m^−1^ s to 75.45 m^−1^ s, 85.42 m^−1^ s, 97.58 m^−1^ s, 89.94 m^−1^ s, 130.54 m^−1^ s, 140.90 m^−1^ s, and 109.83 m^−1^ s by changing the cross-section of *S*_*2*_ from 0.71 m^2^ to 0.73 m^2^, 0.75 m^2^, 0.77 m^2^, 0.79 m^2^, 0.81 m^2^, 0.83 m^2^ and 0.85 m^2^. Besides, *Q* changes from 22,183.31 to 25,879.92, 29,298.19, 33,472.38, 30,849.29, 44,775.59, 48,326.07, and 37,670.87 by changing the cross-section of *S*_*2*_ from 0.71 m^2^ to 0.73 m^2^, 0.75 m^2^, 0.77 m^2^, 0.79 m^2^, 0.81 m^2^, 0.83 m^2^, and 0.85 m^2^. On the other hand, *LoD* changes from 8 × 10^–4^ m s^−1^ to 7 × 10^–4^ 6 × 10^–4^ m s^−1^, 5 × 10^–4^ m s^−1^, 6 × 10^–4^ m s^−1^, 4 × 10^–4^ m s^−1^, 4 × 10^–4^ m s^−1^ and 5 × 10^–4^ m s^−1^ by changing the cross-section of *S*_*2*_ from 0.71 m^2^ to 0.73 m^2^, 0.75 m^2^, 0.77 m^2^, 0.79 m^2^, 0.81 m^2^, 0.83 m^2^ and 0.85 m^2^. As a result, 0.83 m^2^ will be the optimum cross-section.

**Figure 9:**
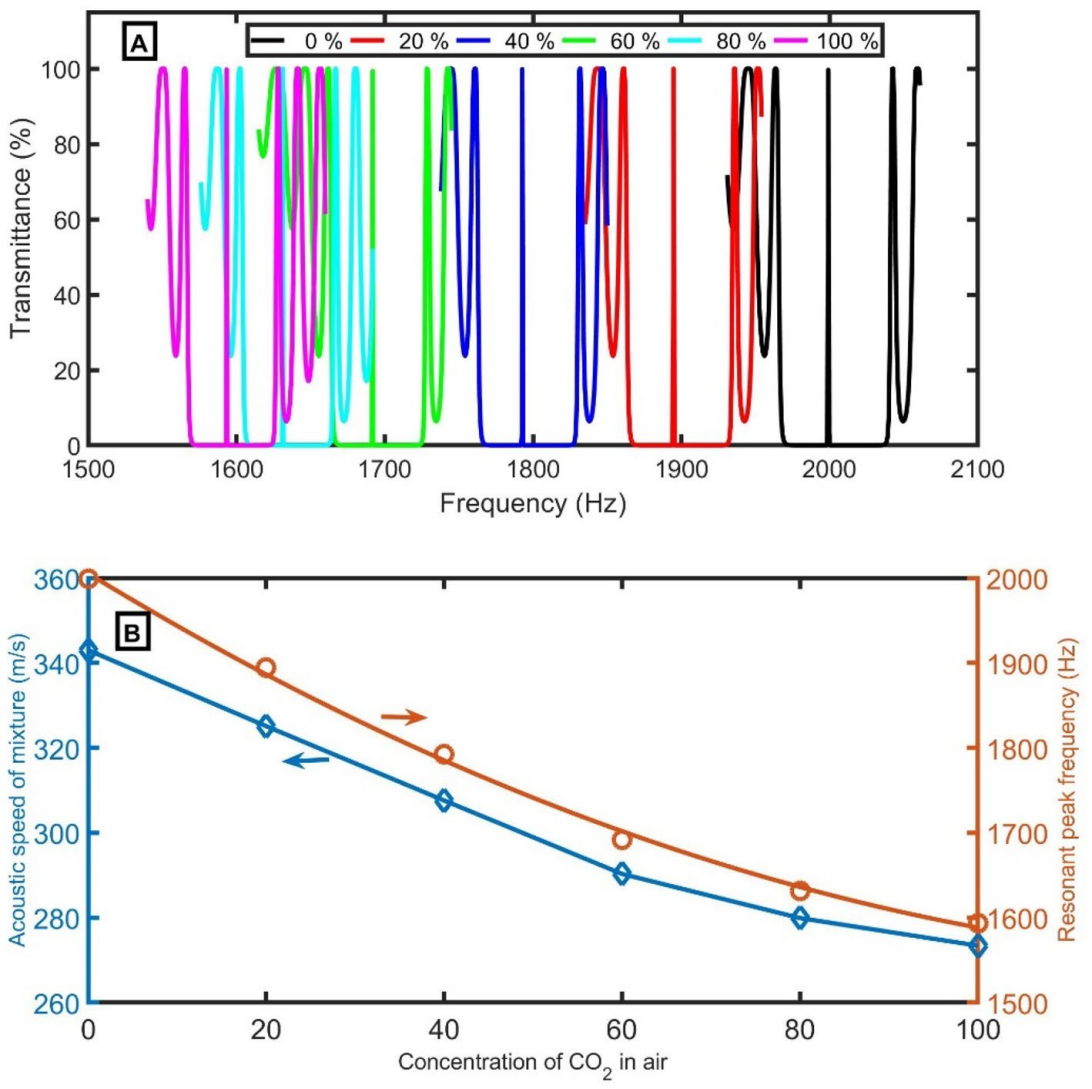
(**A**) The transmittance at selected conditions using air samples with different CO_2_ concentrations, and (**B**) acoustic speed and resonant frequency versus the concentration of CO_2_.

At selected conditions, the defect peak is redshifted to lower frequencies by increasing the concentration of CO_2_ from 1999.02 Hz (at 0% of CO_2_) to 1894.7 Hz (at 20% of CO_2_), 1792.71 Hz (at 40% of CO_2_), 1691.89 Hz (at 60% of CO_2_), 1631.27 Hz (at 80% of CO_2_), and 1593.39 Hz (at 100% of CO_2_), as clear in Fig. 9A. This redshift of the PnBG and resonant peak to lower frequencies is due to the direct proportionality between the acoustic speed of the sample and the resonant frequency according to the standing wave equation:

11 $$2d = \frac{nc}{f},$$

where *d* and *n* are the thickness and an integer, respectively. In Fig. 9B, the acoustic speed and resonant frequency versus the concentration of CO_2_ are plotted. An empirical equation between the resonant frequency ($f_{R}$) and the concentration of CO_2_ ($C_{CO2}$) was established using the quadric fitting as the following relation:

12 $$f_{R} = 0.02222{ }C_{CO2}^{2} - 6.392{ }C_{CO2} + { }2005,{ }\left( {R^{2} = 0.9975} \right).$$

By fitting the simulated data, by knowing the resonant frequency, the CO_2_ concentration can be predicted according to the following equation:

13 $$C_{CO2} = - 1.7656 \times 10^{ - 6} { }f_{R}^{3} + { }0.009803{ }f_{R}^{2} - { }18.299f_{R} + 11511$$

## Conclusion

This study proposed a branched open resonator sensor with a defect guide sandwiched between two PnCs. The structural properties and geometrical parameters of the 1D-PnC sensor composed of branched open resonators were thoroughly optimized. The above simulation studies indicate that the suggested 1D-PnC composed of branched open resonators can effectively detect the concentration of CO_2_ with a sensitivity of 5.8 Hz m^−1^ s, *FoM* of 140 m^−1^.s, *Q* of 5 × 10^4^, and *LoD* of 4 × 10^–4^. Using branched open resonators enhanced the sensor’s performance, according to Table 2. As a result, the suggested design could be useful in different sensing and filtering devices.

**Table 2:** Comparison study.

References	S (Hz s m^−1^)	Q	FoM (s m^−1^)	Structure
2022^33^	2.55	4077	9.16	Binary-asymmetric periodic tubes
2023^34^	1.58	6790	33.7	Ternary-symmetric periodic tubes
This work	5.8	5000	140	Branched open resonator

## Data Availability

Requests for materials should be addressed to Zaky A. Zaky.

## References

[1] Aasi A, Mortazavi B, Panchapakesan B (2022). Two-dimensional PdPS and PdPSe nanosheets: Novel promising sensing platforms for harmful gas molecules. Appl. Surf. Sci..

[2] Boningari T, Smirniotis PG (2016). Impact of nitrogen oxides on the environment and human health: Mn-based materials for the NOx abatement. Curr. Opin. Chem. Eng..

[3] Pham T, Li G, Bekyarova E, Itkis ME, Mulchandani A (2019). MoS2-based optoelectronic gas sensor with sub-parts-per-billion limit of NO2 gas detection. ACS Nano.

[4] Zaky ZA, Al-Dossari M, Matar Z, Aly AH (2022). Effect of geometrical and physical properties of cantor structure for gas sensing applications. Synth. Metals.

[5] Zaky ZA, Amer HA, Suthar B, Aly AH (2022). Gas sensing applications using magnetized cold plasma multilayers. Opt. Quant. Electron..

[6] Roslan NA, Bakar AA, Bawazeer TM, Alsoufi MS, Alsenany N, Majid WHA (2019). Enhancing the performance of vanadyl phthalocyanine-based humidity sensor by varying the thickness. Sens. Actuat. B Chem..

[7] Zayed M, Ahmed AM, Shaban M (2019). Synthesis and characterization of nanoporous ZnO and Pt/ZnO thin films for dye degradation and water splitting applications. Int. J. Hydrogen Energy.

[8] Zaky ZA, Sharma A, Aly AH (2021). Gyroidal graphene for exciting tamm plasmon polariton as refractive index sensor: Theoretical study. Opt. Mater..

[9] Zaky ZA, Aly AH (2021). Gyroidal graphene/porous silicon array for exciting optical Tamm state as optical sensor. Sci. Rep..

[10] Li G, Wang X, Yan L, Wang Y, Zhang Z, Xu J (2019). PdPt bimetal-functionalized SnO_2_ nanosheets: Controllable synthesis and its dual selectivity for detection of carbon monoxide and methane. ACS Appl. Mater. Interfaces..

[11] Mingze L, Jun Z, Molokeev MS, Xingxing J, Zheshuai L, Jing Z (2019). Lead-free hybrid metal halides with a green-emissive [MnBr_4_] unit as a selective turn-on fluorescent sensor for acetone. Inorg. Chem..

[12] Bao Y, Xu P, Cai S, Yu H, Li X (2018). Detection of volatile-organic-compounds (VOCs) in solution using cantilever-based gas sensors. Talanta.

[13] Abd El-Ghany SE, Noum WM, Matar Z, Zaky ZA, Aly AH (2020). Optimized bio-photonic sensor using 1D-photonic crystals as a blood hemoglobin sensor. Phys. Scr..

[14] Khattab MS, Ben-Ali Y, Barkani J, Yousfi J, Bria D (2022). Induced guided acoustic waves in waveguides and resonators. Mater. Today: Proc..

[15] Hu G, Tang L, Liang J, Lan C, Das R (2021). Acoustic-elastic metamaterials and phononic crystals for energy harvesting: A review. Smart Mater. Struct..

[16] Khattab MS, El Kadmiri I, Ben-Ali Y, Khaled A, Jeffali F, Bria D (2023). Propagation of the acoustic waves in a one-dimensional parallel guides and symmetric/asymmetric resonators. Mater. Today: Proc..

[17] Pennec Y, Djafari-Rouhani B, Larabi H, Vasseur J, Hladky-Hennion A (2008). Low-frequency gaps in a phononic crystal constituted of cylindrical dots deposited on a thin homogeneous plate. Phys. Review B.

[18] Alrowaili Z, Aouassa M, Mahmoud M, El-Nasser KS, Elsayed HA, Taha T (2023). Locally resonant porous phononic crystal sensor for heavy metals detection: A new approach of highly sensitive liquid sensors. J. Mol. Liquids.

[19] Lucklum R, Li J (2009). Phononic crystals for liquid sensor applications. Meas. Sci. Technol..

[20] El Boudouti E, Mrabti T, Al-Wahsh H, Djafari-Rouhani B, Akjouj A, Dobrzynski L (2008). Transmission gaps and Fano resonances in an acoustic waveguide: Analytical model. J. Phys. Condens. Matter.

[21] Oseev A, Zubtsov M, Lucklum R (2013). Gasoline properties determination with phononic crystal cavity sensor. Sens. Actuat. B Chem..

[22] Mehaney A (2020). Temperature influences on the performance of biodiesel phononic crystal sensor. Mater. Res. Express.

[23] Antraoui I, Khettabi A (2020). Properties of defect modes in a finite periodic structure with branched open resonators. Mater. Today: Proc..

[24] Zaky ZA, Alamri S, Zhaketov V, Aly AH (2022). Refractive index sensor with magnified resonant signal. Sci. Rep..

[25] Zaky ZA, Singh MR, Aly AH (2022). Tamm resonance excited by different metals and graphene. Photon. Nanostruct. Fundam. Appl..

[26] Meradi KA, Tayeboun F, Guerinik A, Zaky ZA, Aly AH (2022). Optical biosensor based on enhanced surface plasmon resonance: Theoretical optimization. Opt. Quant. Electron..

[27] Zaky ZA, Sharma A, Aly AH (2021). Tamm plasmon polariton as refractive index sensor excited by gyroid metals/porous Ta_2_O_5_ photonic crystal. Plasmonics.

[28] Zaky ZA, Sharma A, Alamri S, Saleh N, Aly AH (2021). Detection of Fat concentration in milk using ternary photonic crystal. SILICON.

[29] Antraoui, I. & Khettabi, A. Defect modes in one-dimensional periodic closed resonators. In *International conference on integrated design and production*, 2019, pp 438–445.

[30] Zaky ZA, Aly AH (2022). Novel smart window using photonic crystal for energy saving. Sci. Rep..

[31] Mehaney A, Ahmed AM (2020). Theoretical design of porous phononic crystal sensor for detecting CO2 pollutions in air. Physica E Low-dimens. Syst. Nanostruct..

[32] Abohassan KM, Ashour HS, Abadla MM (2022). Tunable wide bandstop and narrow bandpass filters based on one-dimensional ternary photonic crystals comprising defects of silver nanoparticles in water. J. Phys. Chem. Solids.

[33] Zaky ZA, Alamri S, Zohny EI, Aly AH (2022). Simulation study of gas sensor using periodic phononic crystal tubes to detect hazardous greenhouse gases. Sci. Rep..

[34] Zaky ZA, Mohaseb M, Aly AH (2023). Detection of hazardous greenhouse gases and chemicals with topological edge state using periodically arranged cross-sections. Phys. Scr..

